# Case Report: Rare case of first-trimester uterine rupture at 9 weeks’ gestation following multiple hysteroscopic surgeries and a prior cesarean section

**DOI:** 10.3389/frph.2026.1812310

**Published:** 2026-04-10

**Authors:** Xiaomin Li, Shouxia Xie, Ling Zhang, Yaying Chu

**Affiliations:** Department of Obstetrics and Gynecology, Jiashan First People’s Hospital, Jiaxing, China

**Keywords:** acute and critical illness, early pregnancy, hemorrhagic shock, intrauterine adhesions, uterine rupture

## Abstract

Uterine rupture is a rare but life-threatening obstetric emergency, with most cases occurring during late pregnancy or labor, particularly in patients with a history of uterine surgery. This report presents an exceptionally rare case of spontaneous uterine rupture at 9 weeks' gestation in a 36-year-old woman with a complex surgical history, including a midtrimester cesarean delivery for placenta accreta and multiple subsequent hysteroscopic adhesiolysis procedures. The patient presented with acute abdominal pain and hemorrhagic shock, highlighting the diagnostic challenges of early gestational rupture, in which ultrasonographic findings may be nonspecific. Despite initially inconclusive imaging, prompt laparoscopic intervention confirmed a 4-cm anterior uterine wall rupture with significant hemoperitoneum, enabling successful laparoscopic repair and preservation of reproductive potential. This case highlights several critical clinical insights, including the potential cumulative weakening effect of multiple uterine interventions beyond classical cesarean scarring, feasibility of minimally invasive management in hemodynamically stable patients, and importance of maintaining a high index of suspicion in high-risk populations regardless of gestational age. This atypical presentation challenges conventional understanding of uterine rupture timelines and risk stratification, emphasizing the need for individualized assessments of uterine integrity in patients with complex surgical histories. This report contributes to the limited literature on first-trimester uterine rupture and underscores the evolving paradigm of diagnostic and therapeutic approaches for this catastrophic obstetric complication.

## Introduction

Uterine rupture is a life-threatening obstetric emergency characterized by a full-thickness disruption of the uterine wall, typically occurring during late pregnancy or labor (1). While its incidence of unscarred uteri is exceptionally low (0.2%), the risk increases to 3.8%–4.3% in patients with a history of uterine surgery, making prior cesarean delivery the predominant risk factor (2). The pathophysiology involves mechanical stress exceeding the tensile strength of compromised myometrial tissue, particularly at sites of previous surgical scarring or congenital anomalies (3). Spontaneous rupture in early pregnancy (≤12 weeks) is exceedingly rare, with most reported cases occurring in patients with scarred uteri or structural abnormalities, such as adenomyosis or Müllerian duct anomalies (4).

The diagnostic challenge of first-trimester uterine rupture stems from its nonspecific presentation, often mimicking other acute abdominal conditions, such as ectopic pregnancy or appendicitis (5). Unlike classical third-trimester rupture, which typically manifests as fetal distress and hemorrhagic shock, early gestational rupture may present with subtle symptoms until catastrophic hemorrhage occurs (6). Ultrasonography, although the first-line imaging modality, has limited sensitivity for detecting small uterine defects in early pregnancy, with reported misdiagnosis rates of up to 30% (7). This limitation underscores the critical role of heightened clinical suspicion in high-risk populations, particularly patients with prior uterine interventions.

The present case is clinically significant because of the convergence of multiple high-risk features: (1) rupture at 9 weeks' gestation, preceding the period of substantial uterine distension; (2) a complex surgical history, including midtrimester cesarean delivery for placenta accreta followed by multiple hysteroscopic adhesiolysis procedures; and (3) absence of classical ultrasonographic findings despite massive hemoperitoneum (8). Such cases challenge conventional assumptions regarding uterine rupture timelines and risk stratification models. Furthermore, successful laparoscopic management in this case demonstrates the feasibility of minimally invasive approaches in carefully selected hemodynamically stable patients, in contrast to traditional emergency laparotomy paradigms (9).

This case provides valuable insights into several underexplored aspects of uterine rupture, including the potential role of Asherman syndrome in compromising myometrial integrity (10), limits of hemodynamic compensation during early pregnancy hemorrhage (11) and the importance of multidisciplinary rapid-response systems in obstetric emergencies (12). By describing this exceptional presentation, we aim to improve recognition of atypical uterine rupture patterns and optimize management strategies for this catastrophic complication across all gestational ages.

## Case presentation

### Patient information

A 36-year-old woman, gravida 2, para 0, presented to the emergency department on the night of December 27, 2024, with a chief complaint of acute-onset nausea, vomiting, and lower abdominal pain lasting more than 1 h. The patient was at 9 weeks' gestation, with the last menstrual period dated October 29, 2024. Pregnancy had been confirmed, and a transvaginal ultrasound performed 9 d earlier demonstrated an intrauterine pregnancy corresponding to approximately 7 weeks and 6 days of gestation, with a 13-mm embryo and visible cardiac activity, along with a small amount of intrauterine fluid measuring 14 × 4 mm. The patient was receiving daily intramuscular progesterone injections (20 mg) for luteal phase support. On the day of admission, approximately 1 h after experiencing nausea and a single episode of nonprojectile, nonbilious, nonbloody vomiting, the patient developed severe cramping lower abdominal pain accompanied by chills and dizziness. No vaginal bleeding or leakage of fluid was reported. The patient's medical history was significant for a prior pregnancy at 25 weeks' gestation in 2020, complicated by placenta accreta and massive hemorrhage during induction of labor, necessitating emergency laparotomy for fetal extraction and subsequent blood transfusion. Following this event, the patient developed secondary amenorrhea and was diagnosed with intrauterine adhesions (Asherman syndrome). Over the subsequent 4 years, the patient underwent multiple hysteroscopic adhesiolysis procedures. Menstruation resumed in March 2024, although flow remained scant and was not associated with dysmenorrhea.

### Clinical findings

On admission, the patient was pale, alert, and distressed. Vital signs were notable for hypotension (blood pressure of 70/50 mmHg), with a pulse of 80 beats per minute, respiratory rate of 20 breaths per minute, and temperature of 36.9 °C. An abdominal examination revealed a soft abdomen with tenderness and guarding in the lower quadrants, without significant rebound tenderness. A 12-cm vertical surgical scar was observed in the suprapubic region. Bowel sounds were present, and shifting dullness was positive. Pelvic examination revealed a closed cervical os with no active bleeding. The uterus was anteverted, enlarged to the size consistent with an 8-week gestation, and tender. Cervical motion tenderness was noted. The left adnexa was thickened and tender, whereas the right adnexa was mildly tender without palpable masses. Culdocentesis yielded 5 mL of dark red, nonclotted blood. Emergency bedside ultrasonography demonstrated an anteverted uterus corresponding to an 8-week gestational size, with an intact serosal contour. An intrauterine gestational sac containing a 19-mm embryo without detectable cardiac activity was observed. The myometrial echotexture appeared homogeneous. Neither ovary was visualized. A significant amount of free fluid with poor echogenicity, measuring approximately 95 × 55 mm, was observed in the pelvis. A subsequent transabdominal scan confirmed the presence of free intraperitoneal fluid at a maximum depth of approximately 32 mm in the hepatorenal recess. Blood chorionic gonadotropin *β* (β-hCG) 27530.6 mIU/mL, Hemoglobin level 105 g/L, hematocrit 0.316 V/V, platelet count 275*10 ^ 9/L, lactate level 3.4 mmol/L. In addition, the coagulation function is within the normal range.

### Diagnostic assessment

The initial clinical impression, based on the acute presentation of severe abdominal pain, hypotension, positive culdocentesis, and ultrasonographic findings of massive hemoperitoneum in a patient with a history of uterine surgery, was acute intra-abdominal hemorrhage suspected to be caused by uterine rupture. The differential diagnoses included other causes of hemoperitoneum in early pregnancy, such as ruptured ectopic pregnancy or bleeding from a corpus luteum cyst. However, the confirmed intrauterine gestation and patient's surgical history made uterine rupture the primary concern. The final diagnosis of complete uterine rupture was confirmed intraoperatively. This diagnostic assessment underscores the critical importance of maintaining a high index of suspicion in patients with risk factors presenting with acute abdominal pain and shock in early pregnancy, utilizing a combination of clinical evaluation, focused ultrasonography, and diagnostic procedures, such as culdocentesis, to expedite surgical intervention.

### Therapeutic intervention

**Figure 1:**
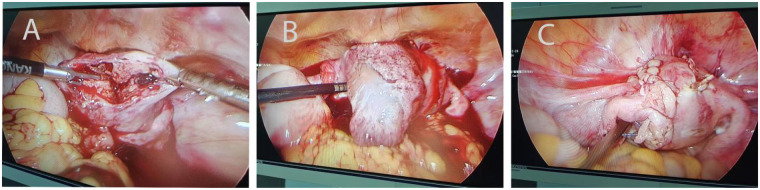
Images observed during emergency laparoscopic exploration. **(A)** An irregular longitudinal laceration, approximately 4.0 cm long, was observed on the left side of the mid-segment of the anterior wall of the uterus. **(B)** The lacerated surface was adherent with chorionic villi tissue and accompanied by active bleeding. **(C)** Laparoscopic repair of the uterine rupture was performed.

The patient was immediately resuscitated with intravenous fluids, and blood products were cross-matched. Given the diagnosis of hemorrhagic shock and suspected uterine rupture, emergency diagnostic laparoscopy was performed. The procedure revealed approximately 2,000 mL of dark red blood and clots within the peritoneal cavity. A complete, vertical, and irregular rupture approximately 4.0 cm in length was identified on the anterior uterine wall, extending from near the fundus to the vesicouterine peritoneal reflection (Figure 1A). Active bleeding was noted at the rupture site, which was adherent to the chorionic villi (Figure 1B). The left fallopian tube demonstrated hydrosalpinx and was adherent to the anterior peritoneum. Laparoscopic repair of the uterine rupture was performed (Figure 1C). The chorionic tissue was carefully extracted from the rupture site using spoon forceps and removed using an endoscopic retrieval bag. Hemostasis was achieved by injecting 6 IU of vasopressin into the myometrium. The uterine defect was closed in two layers using a 2–0 absorbable suture. Following the repair, suction curettage was performed under laparoscopic visualization to ensure complete evacuation of the uterine cavity. A pelvic drain was placed at the conclusion of the surgery. Intraoperative blood loss was estimated to be 2,000 mL, necessitating transfusion of 3 units of O Rh-positive packed red blood cells. Postoperative management included intravenous antibiotics (clindamycin 0.6 g twice daily) for infection prophylaxis and oxytocin (20 units daily) to promote uterine contraction.

### Follow-up and outcomes

The patient's postoperative recovery was uneventful. The patient was discharged on postoperative day 5 following suture removal. At the 2-month follow-up visit, menstruation had resumed, although the flow remained scant. Follow-up transvaginal ultrasonography revealed no significant abnormalities. The patient was advised to practice strict contraception for a minimum of 2 years and will continue to be monitored in a long-term follow-up program to assess future reproductive potential and provide counseling regarding the significantly elevated risks associated with any subsequent pregnancy (Table 1).

**Table 1:** Timeline of key clinical events and interventions.

Date/Time	Event	Key Data/Findings
October 29, 2024	Last menstrual period	Gestational age: 0 weeks
December 18, 2024	Transvaginal ultrasound (routine follow-up)	Intrauterine pregnancy, 7 weeks 6 days; embryo 13 mm with cardiac activity; small intrauterine fluid (14 × 4 mm)
December 27, 2024 (Evening)	Onset of symptoms: nausea, vomiting, lower abdominal pain, dizziness	No vaginal bleeding; onset 1 h after vomiting
December 27, 2024 (Admission)	Emergency department presentation	Blood pressure 70/50 mmHg, pulse 80 bpm; positive culdocentesis; free fluid in pelvis (95 × 55 mm) and hepatorenal recess (32 mm); *β*-hCG 27530.6 mIU/mL, Hemoglobin level 105 g/L, hematocrit 0.316 V/V, platelet count 275*10 ^ 9/L, lactate level 3.4 mmol/L
Immediate post-admission	Emergency bedside ultrasound	Intrauterine pregnancy, embryo 19 mm, no cardiac activity; intact serosal contour; massive hemoperitoneum
Same night	Emergency diagnostic laparoscopy	2,000 mL hemoperitoneum; 4 cm anterior uterine wall rupture; active bleeding; chorionic villi adherent
Intraoperative	Laparoscopic repair and suction curettage	Uterine defect closed in two layers; 3 units PRBC transfused; estimated blood loss: 2,000 mL
Postoperative Day 1–5	Inpatient recovery	IV antibiotics (clindamycin), oxytocin; pelvic drain removed; stable recovery
Postoperative Day 5	Discharge	Suture removal; no complications
2-month follow-up	Outpatient visit and transvaginal ultrasound	Menstruation resumed (scant flow); no significant abnormalities; contraception advised for ≥2 years

## Discussion

This case presents a rare occurrence of spontaneous uterine rupture at 9 weeks of gestation, whereas uterine rupture is typically reported in the second or third trimester in the literature (1). While studies have identified cesarean section as the predominant risk factor, accounting for 45%–69.74% of reported cases (13), our patient exhibited a unique combination of prior midtrimester placental implantation requiring cesarean delivery and subsequent multiple hysteroscopic adhesiolysis procedures, a clinical profile that is scarcely documented in existing reports (10). Notably, the rupture site in this case—the anterior uterine body—contrasted with the fundal predominance (43%) and lower-segment localization (44%) described in systematic reviews (6), suggesting an altered distribution of biomechanical stress may due to iatrogenic adhesions. Zhang et al. (14) reported a case of spontaneous rupture of a scar free uterus in early pregnancy, which was different from our report. The report found that it was caused by complete placenta previa during surgery.

The diagnostic challenges observed in this case align with literature emphasizing the limitations of ultrasonography in detecting early gestational uterine rupture (4); however, this case demonstrates more timely diagnostic confirmation through immediate laparoscopic exploration, compared with the 61% of reported cases in which diagnosis required surgical confirmation after delayed recognition (6). Unlike the 30% hysterectomy rate reported among patients with first-trimester uterine rupture (6), successful laparoscopic repair in this case highlights the evolving role of minimally invasive management. Furthermore, the absence of placental accreta-a condition reported in 43.7% of midgestational uterine ruptures (1) underscores the critical contribution of structural uterine compromise resulting from repeated instrumentation. These distinctions emphasize the need for heightened vigilance in patients with complex surgical histories beyond classical cesarean scarring.

This case highlights several critical considerations related to the diagnostic pitfalls and pathophysiological mechanisms of early uterine rupture. The initial ultrasonographic examination failed to detect rupture, a common diagnostic challenge in early gestation, in which small myometrial defects may be obscured by the gravid uterus and intrauterine contents. This finding underscores the importance of correlating imaging results with clinical status; in the presence of hemorrhagic shock and positive culdocentesis, reliance on ultrasonography alone would have been dangerously misleading. The decision to proceed with diagnostic laparoscopy was pivotal, serving as both a definitive diagnostic modality and therapeutic intervention, thereby averting a potentially catastrophic delay. This approach aligns with the evolving paradigm that minimally invasive surgery can be safely employed in acute hemorrhagic settings, provided that hemodynamic stability is maintained through aggressive resuscitation.

The pathological mechanism in this patient represents an instructive deviation from the classic model of isolated scar dehiscence. While a prior cesarean scar is a well-established risk factor, this case illustrates a compounded vulnerability. The original scar from cesarean delivery for placental implantation likely created a focal area of structural weakness, which was exacerbated by multiple hysteroscopic adhesiolysis procedures. These subsequent interventions, while restoring menstrual function, may have induced microtrauma, fibrosis, and aberrant myometrial healing, thereby critically compromising tensile strength (15). This sequence of events created a biomechanical environment in which the combined effects of focal scarring and diffuse myometrial disruption predisposed the patient to rupture at an unusually early gestational age and at a site atypical for classical lower-segment rupture. This multifactorial etiology underscores that uterine integrity exists along a continuum and that a history of uterine surgery not limited to cesarean section alone warrants careful risk stratification (16). The patient's uneventful recovery and resumption of menstruation further confirm that, with prompt diagnosis and meticulous surgical repair, fertility preservation remains a viable and essential goal, even in the setting of significant hemorrhage (17).

This case provides valuable clinical insights while highlighting important limitations. Successful laparoscopic management challenges traditional paradigms favoring emergency laparotomy, demonstrating that minimally invasive approaches are feasible in selected hemodynamically stable patients. However, this experience also underscores the need for individualized decision-making, as delayed conversion to laparotomy in unstable patients could prove catastrophic. This case serves as a critical reminder that uterine rupture should be included in the differential diagnosis of acute abdominal pain in early pregnancy, particularly in patients with complex uterine surgical histories beyond classical cesarean scarring.

This study has several limitations that warrant consideration. Firstly, a follow-up period of 2 months is too short to assess reproductive outcomes in the later stages. The American College of Obstetricians and Gynecologists and multiple studies support using 18–24 months as an ideal pregnancy interval to promote good healing of uterine scars and maternal recovery, significantly reducing the risk of uterine rupture in pregnant women (18, 19). Because this report describes a single-center case, the generalizability of the findings is limited. Long-term reproductive outcomes following laparoscopic repair remain uncertain, necessitating further studies with larger cohorts and extended follow-up. Additionally, although this case suggests that multiple uterine surgeries may synergistically increase rupture risk, the relative contribution of each procedure requires validation through systematic investigation. These gaps emphasize the need for multicenter collaboration to establish evidence-based guidelines for monitoring and managing high-risk pregnancies in patients with complex uterine surgical histories.

## Data Availability

The original contributions presented in the study are included in the article/Supplementary Material, further inquiries can be directed to the corresponding author.
